# Supplementary material for: Health-related quality of life and its predictors among chronic kidney disease patients: A hospital-based cross-sectional study
Source: PLoS One. 2025 Feb 7;20(2):e0319100. doi: 10.1371/journal.pone.0319100 (PMC11805394; doi:10.1371/journal.pone.0319100)
Supplement: Attachment — Submitted filename: Recommendations.docx [file pone.0319100.s002.docx]

It would be informative and useful to narrate in introduction, discussion and conclusion how would this study outcome can be translated into focused tailoring of the treatment plan, bench to bedside. There should be some suggestions for future studies based on the outcome of this study.
